# Experimental Study on Nano-Parameters of Styrene-Butadiene-Styrene Block Copolymer Modified Bitumen Based on Atomic Force Microscopy

**DOI:** 10.3390/polym11060989

**Published:** 2019-06-04

**Authors:** Wengang Zhang, Fang Wang, Jun Shi, Zhongmei Li, Xiaofei Liang

**Affiliations:** 1 School of Civil and Architectural Engineering, Shandong University of Technology, Zibo 255049, China; 2 School of Physics and Optoelectronic Engineering, Shandong University of Technology, Zibo 255049, China; wangfangsdut@163.com

**Keywords:** SBS modified bitumen, nano-parameters, atomic force microscope, pavement performances

## Abstract

In this paper, an atomic force microscope (AFM) was used to study the nano-morphology characteristics of styrene-butadiene-styrene block copolymer (SBS) modified bitumen. The effects of SBS content and short-term aging on the nano-morphology parameters of bitumen were studied. The correlation between nano-morphology parameters and main pavement performances of SBS modified bitumen was analyzed. The results show that with the increase of SBS content, the average area of bee-like structure and the proportions of bee-like structure area of SBS modified bitumen increases gradually, the roughness of the whole image and the roughness of the lightweight component region decreases gradually, but the increasing or decreasing trend is no longer obvious when the content of SBS is more than 4%. The maximum height difference of SBS modified bitumen is linear with the content of SBS. The larger the content of SBS, the smaller the influence of short-term aging on the nano-morphology of SBS modified bitumen. The penetration degree will decrease and the softening point will increase with the increase of the proportion of bee-like structure area of SBS modified bitumen. The ductility will decrease with the increase of the roughness of the lightweight component region. The ductility and the Brookfield viscosity will increase with the increase of the maximum height difference. The research results provided a reference for defining quantifiable nano-parameters of SBS modified bitumen and establishing the relationship between nano-parameters and pavement performances.

## 1. Introduction

At present, styrene-butadiene-styrene block copolymer (SBS) modified bitumen is the most widely used modified bitumen in the world, and the market share is more than 95% [1,2,3]. The specifications of SBS-modified bitumen has been established, which used viscosity as the key index, low-temperature index, fatigue index, temperature sensitivity, durability, adhesiveness, and viscoelasticity as the auxiliary performance system and corresponding evaluation index system [4,5]. However, this still cannot guarantee the good performance of SBS modified bitumen in the process of use, so researchers try to study SBS modified bitumen from the micro and nano perspective [6,7,8]. In recent years, micro-detection technology represented by fluorescence microscope and nano-detection technology represented by atomic force microscope (AFM) has been applied in the detection of SBS modified bitumen [9,10,11]. When the short light wave from the fluorescence microscope irradiates the bitumen sample film, the color of SBS is yellow, while bitumen and other substances are basically black, therefore, the two-dimensional micro-images of SBS modified bitumen can be obtained by fluorescence microscopy. It is generally believed that the area ratio and structural fineness of SBS in fluorescence microscopic images have significant effects on the technical performance of bitumen [12,13,14]. AFM is an analytical instrument which can be used to study the surface structure of SBS modified bitumen. The surface structure and properties of bitumen can be studied by detecting the very weak interaction force between the bitumen surface and a micro force sensor [15]. Fixed one end of the micro-cantilever, which is extremely sensitive to weak force, the micro-needle tip at the other end approaches the bitumen, the force will make the micro-cantilever deformation or motion state change, these changes can be detected by the sensor, and the force distribution information are obtained, then the surface topography structure information and the surface roughness information can be obtained at a nanometer resolution. Compared with fluorescence microscopy, the imaging accuracy of AFM is higher, and the three-dimensional image is more intuitive [16,17,18,19,20].

In 1996, the bee-like structure in bitumen was first observed by Loeber with AFM [11]. The current mainstream view held that the existence of asphaltene was the most important factor affecting the morphology of bitumen under AFM [21,22,23,24,25,26]. About the location and conditions of the bee-like structure exist, Ramm et al. used non-contact optical microscopy and optical scattering technology to observe that the bee-like structure of bitumen existed only on the surface and did not appear immediately during the heating-cooling cycle [27]. De Moraes et al. [28] found that when the temperature of bitumen exceeded 57 °C, the bee-like structure would disappear. These changes are related to the component changes in bitumen aging process, Wu Shaopeng et al. [29] reported that With the oxidation of base bitumen and the degradation of SBS segments, obvious asphaltene micelles appeared in both binders. Many researchers had tried to establish the relationship between nano-parameters and aging performance, including the reduction of bee-like structure and the degree of aggregation after aging [30]. With the help of AFM, Dehouche et al. [31] finished a study on the uniformity of organic montmorillonite modified bitumen. Zhang et al. [32] reported that with the increase of organic montmorillonite/SBS composite modifier content, the dispersed phase area of bitumen increased. From the AFM phase diagram of SBS modified bitumen, Li bo’s team found that the continuous phase area of lightweight components of bitumen decreased with the SBS content increase [33]. Yang Zhen et al. [34] reported that the micromechanics of the asphalt surface contribute to the macro-mechanical behavior. Zhang Henglong et al. [35] reported that the aging affected the bitumen morphology significantly, aging increased the overall surface stiffness of the bitumen and made the bitumen surface more solid-like. AFM technology has become an important means to study bitumen and modified bitumen, some progress has been made in bitumen nano-morphology testing methods and influencing factors of morphology change, but the main problems are yet to be solved. They are:

(1) The first problem is the lack of quantifiable nano-parameters of SBS modified bitumen.
(2) The second problem is that the relationship between the nano-parameters of SBS modified bitumen and its pavement performances is still unknown.

In this paper, modified bitumen with different SBS content was prepared, and the nano-parameters were obtained by AFM at room temperature, such as, the average area of the bee-like structure, the proportion of bee-like structure area, the roughness of the whole image, the roughness of the lightweight component region and the maximum height difference. The relationships of nano-parameters with SBS content and short-term aging were studied. The correlation between the nano-parameters and main pavement performances of SBS modified bitumen was analyzed. The technical means of this paper are relatively novel, such as the measurement of the bee-like structure area and the maximum height difference. Up to now, the roughness of different phases of bitumen in this paper was tested for the first time. The research results provided a reference for defining quantifiable nano-parameters of SBS modified bitumen and establishing the relationship between nano-parameters and pavement performances.

## 2. AFM Test of SBS Modified Bitumen

### 2.1. Preparation of SBS Modified Bitumen

**Figure 1:**
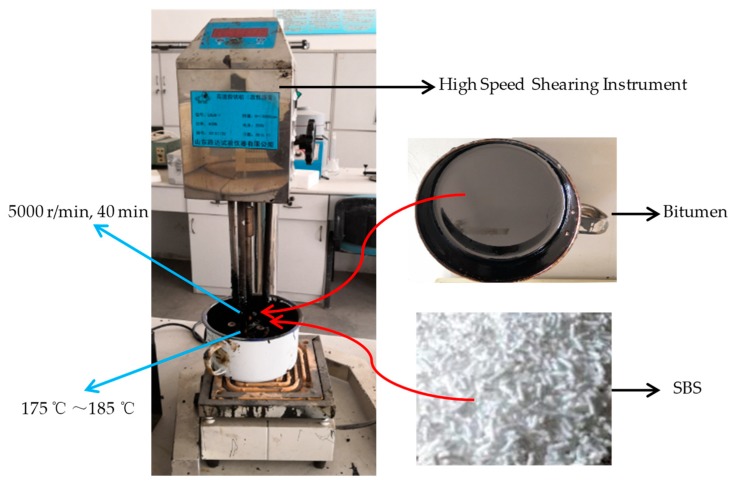
Preparing of SBS modified bitumen.

A-70 bitumen used in this paper was produced by Sinopec Qilu Petrochemical Company (Zibo, China). The technical index is shown in Table 1. SBS used in this paper was produced by Sinopec Yanshan Petrochemical Company (Beijing, China). The SBS modified bitumen was prepared by the following process: firstly, the bitumen was heated to 175 °C and mixed with SBS modifier. Secondly, the bitumen was sheared at 5000 r/min for 40 min by using a high-speed shearing device [36], the temperature was kept between 175 °C and 185 °C during shearing. Finally, SBS modified bitumen was put into the oven at 165 °C for 2 h. The preparation process of SBS modified bitumen is shown in Figure 1.

The aged SBS modified bitumen can be obtained by rolling thin-film oven test (RTFOT), the test condition was 163 ± 0.5 °C, 85 min.

### 2.2. Preparation of AFM Samples

**Figure 2:**
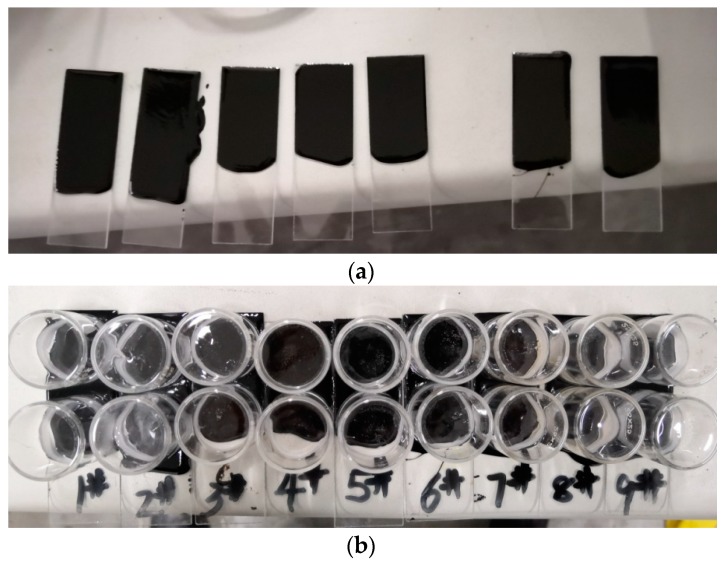
Photographs of AFM sample preparation ((**a**) keep the sample in a horizontal state; (**b**) dust-proof).

SBS modified bitumen was dripped on the slide, then heated briefly to make the bitumen lie flat, and placed on the horizontal table quickly. After cooling for 15 min, the surface of bitumen was covered with a glass container to prevent sample pollution [32]. Figure 2 is the photographs of the preparation of the AFM samples.

### 2.3. AFM Test

The atomic force microscope (Bruker Dimension Icon) used in this paper was produced by Bruker Company (Rheinstetten, Germany). The test conditions of AFM are shown in Table 2.

**Figure 3:**
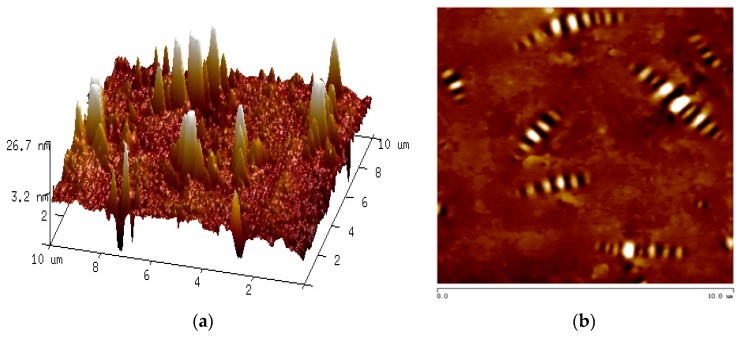
AFM images of bitumen ((**a**) three-dimensional height image; (**b**) two-dimensional height image).

Figure 3 below is an example of the AFM images of bitumen.

Figure 3 shows that the surface of the bitumen is not flat. The two-dimensional height image shows that there is much bee-like structure which similar to the body of bees on the surface of bitumen. Many nano-parameters of bitumen, such as the average area of the bee-like structure, the proportion of bee-like structure area, the roughness of the whole image, the roughness of the lightweight component region and the maximum height difference, can be obtained from the AFM images. In order to reduce the error, at least six scanning points were selected for each test sample.

## 3. Nanoscopic Morphology Analysis of SBS Modified Bitumen at Room Temperature

### 3.1. Nanoscopic Morphology of Modified Bitumen with Different SBS Content

**Figure 4:**
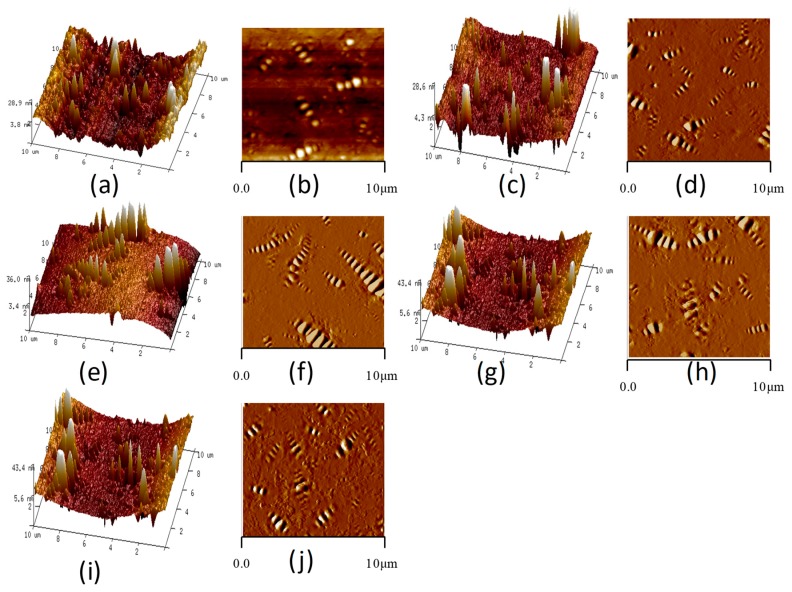
AFM images of bitumen with different SBS content (**a**,**c**,**e**,**g**,**i** are the three-dimensional height images, the content of SBS are 0, 3%, 4%, 5%, and 6%, respectively; **b**,**d**,**f**,**h**,**j** are the two-dimensional height images, the content of SBS are 0, 3%, 4%, 5%, and 6%, respectively).

In this paper, the nano-morphology of modified bitumen with different SBS content was tested by AFM. The SBS content was 0, 3%, 4%, 5%, and 6% respectively, and the test temperature was 25 °C. Figure 4 shows the AFM images of bitumen with different SBS content.

#### 3.1.1. Area of Bee-Like Structure

**Figure 5:**
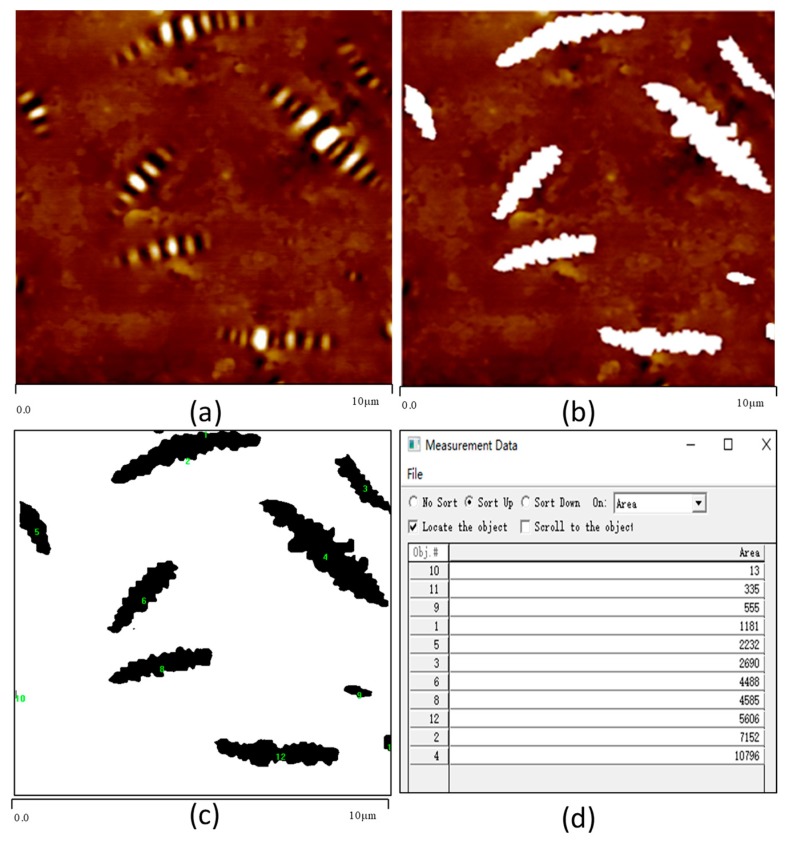
Measurement of the bee-like structure area ((**a**) two-dimensional height image; (**b**) two-dimensional height image without bee-like structure; (**c**) the measurement of each bee-like structure area; (**d**) the measurement results of the bee-like structure area).

**Figure 6:**
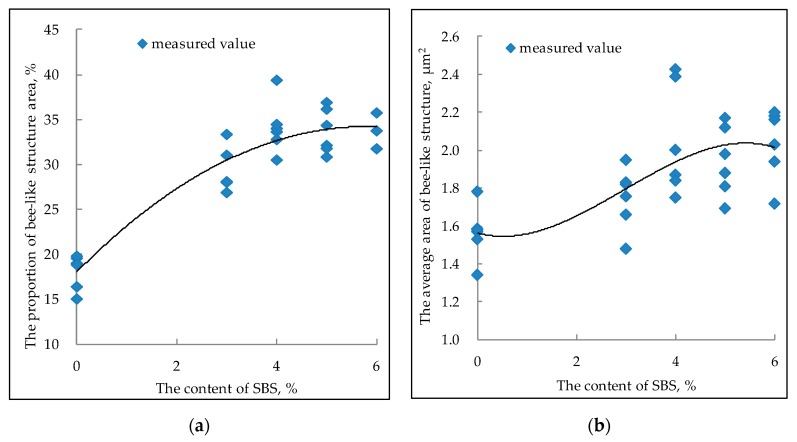
Relationship between SBS content and bee-like structure area ((**a**) the proportion of bee-like structure area; (**b**) the average area of bee-like structure).

Figure 4 shows that with the increase of SBS content, the shape of bee-like structure changes. The number of the bee-like structure increases first and then decreases, and the shape of the bee-like structure is gradually slender. The two-dimensional height image of the bee-like structure of bitumen has obvious boundaries, which can be used for image matting by Photoshop software, and Image-Pro-Plus software can be used to measure each bee-like structure area and the total area. As shown in Figure 5. Then the proportion of bee-like structure area and the average area of bee-like structure can be calculated. The proportion of bee-like structure area and the average area of the bee-like structure of modified bitumen with different SBS content are shown in Figure 6.

Figure 6 shows that with the increase of the SBS content, the area of the bee-like structure of SBS modified bitumen increases gradually and then tends to be stable. Especially when the content of SBS is more than 4%, the increasing trend is no longer obvious. The content of SBS has a similar relationship with the proportion of bee-like structure area. The absorption of light components in bitumen by SBS increases the proportion of asphaltene in bitumen and aggregation of asphaltene, while the bee-like structure is mainly due to the existence of asphaltene. Therefore, the average area and the proportion of bee-like structure in the AMF image of SBS modified bitumen increase [34].

#### 3.1.2. Roughness

**Figure 7:**
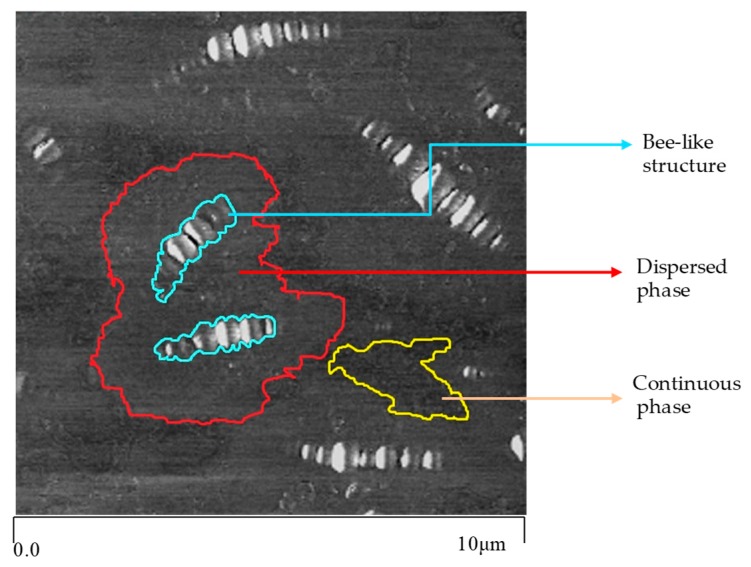
Different phases in the AFM phase image of SBS modified bitumen.

**Figure 8:**
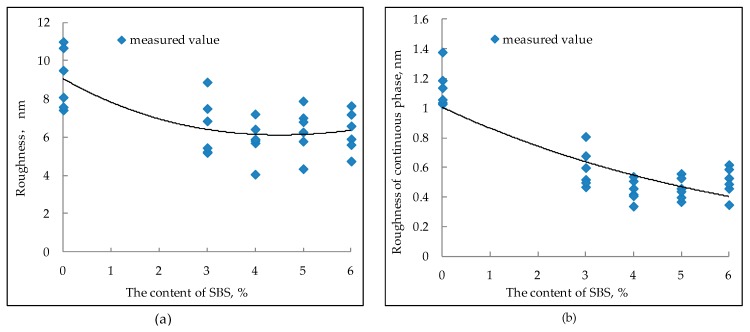
Relationship between roughness and the content of SBS modifier ((**a**) the roughness of the whole image; (**b**) the roughness of continuous phase).

The boundaries between different phases in the phase image are clear, so the AFM phase image of SBS modified bitumen can be divided into three parts: the bee-like structure, the dispersed phase and the continuous phase [11], in which the continuous phase is light oil composed of aromatic and saturated components of bitumen as shown in Figure 7. In addition, roughness can reflect the fluctuation degree of bitumen surface to a certain extent, and the roughnesses of different phases with different fluctuations are also different. With the help of Nanoscope Analysis Software, the roughness of AFM images of bitumen can be read directly, and the roughness of continuous phase (light oil) of bitumen can also be read directly too. The relationship between roughness and the content of SBS modifier is shown in Figure 8.

Figure 8 shows that when the SBS content is 0%, the roughness of the whole image and the roughness of the continuous phase is bigger than other SBS contents. With the increase of SBS content, the roughness of the whole image and the roughness of the continuous phase show a decreasing trend, but when the SBS content exceeds 4%, the decreasing trend is no longer obvious, and even there is a slight upward trend. Compared with the SBS content of 0%, the reduction ratio of roughness when SBS content is 3%, 4%, 5%, and 6% is 29.2%, 32.1%, 32.2%, and 30.1%, respectively, for continuous phase, the values were 48.6%, 58.4%, 64.1%, and 54.5%. The reason for the decrease of roughness is mainly due to the absorption of light components by SBS. The increase of SBS content can also lead to an increase of bee-like structure volume and area proportion, which can affect the roughness of bitumen to a certain extent. This is also the reason why the roughness of bitumen increases slightly after the SBS content exceeds 4%.

#### 3.1.3. Maximum Height Difference

**Figure 9:**
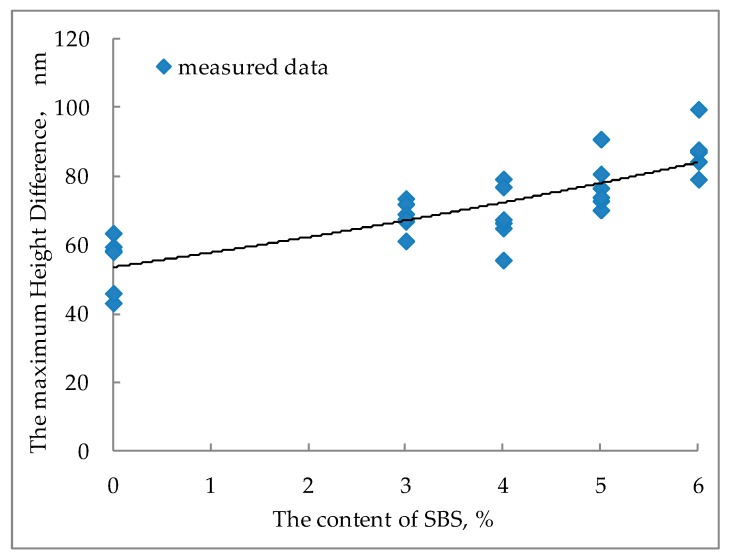
Maximum height difference of modified bitumen with different SBS content.

The maximum height difference in the AFM image of bitumen refers to the difference between the highest point and the lowest point of the bitumen surface [24]. The maximum height difference can reflect the fluctuation degree of the bitumen surface to a certain extent. Figure 9 shows the maximum height difference of modified bitumen with different SBS content.

It can be seen from Figure 9 that the maximum height difference in the AFM image increases linearly with the increase of SBS content. The reason for this rule is that the increase of SBS content absorbs more light components, resulting in a significant decrease in the continuous phase of SBS modified bitumen, agglomeration of asphaltenes, and an increase in the undulation of bee-like structures. In addition, this phenomenon is also well presented in the three-dimensional images of SBS modified bitumen in Figure 4.

### 3.2. Nanoscopic Morphology of Modified Bitumen after Aging

**Figure 10:**
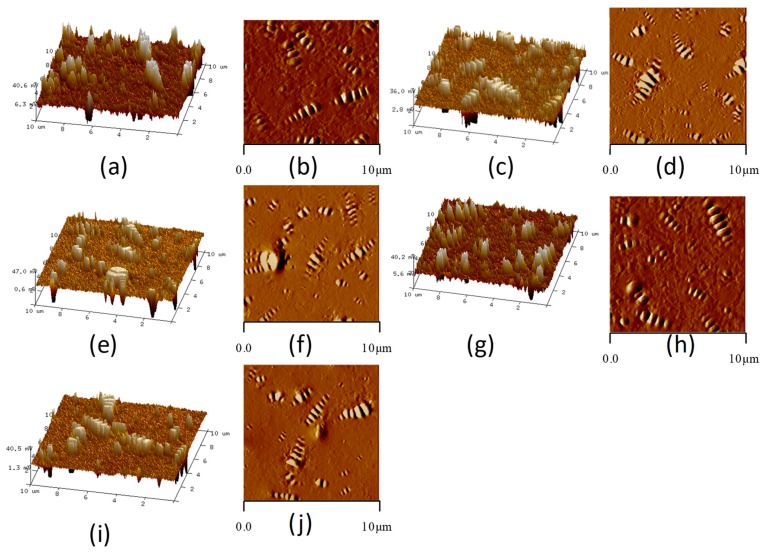
AFM images of bitumen with different SBS contents after aging (**a**,**c**,**e**,**g**,**i** are the three-dimensional height images, the content of SBS are 0, 3%, 4%, 5%, and 6%, respectively; **b**,**d**,**f**,**h**,**j** are the two-dimensional height images, the content of SBS are 0, 3%, 4%, 5%, and 6%, respectively).

**Figure 11:**
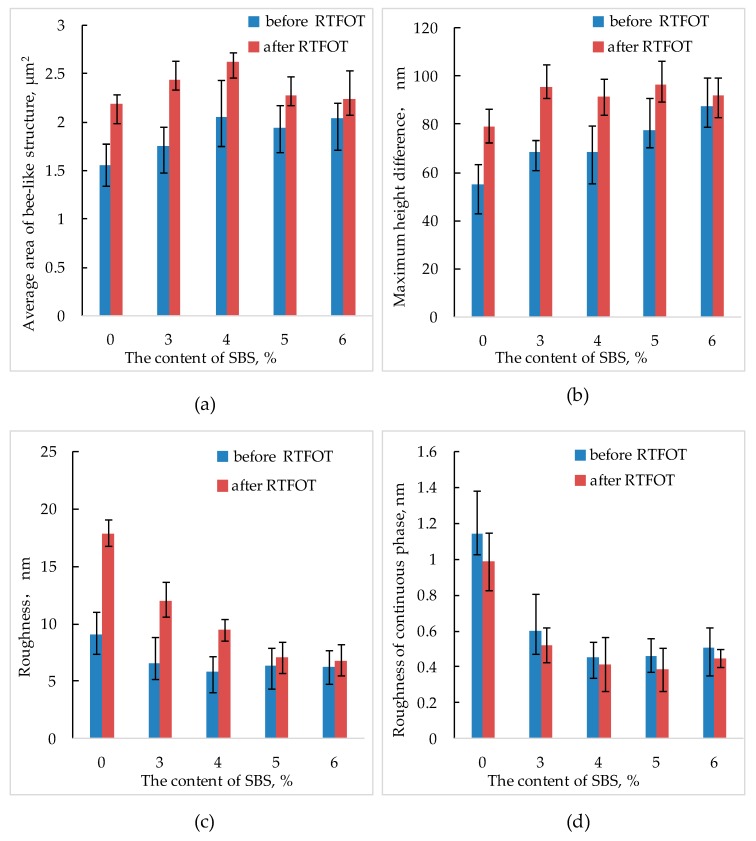
Comparison of the nano-parameters of SBS modified bitumen before and after RTFOT under different SBS content ((**a**) the average area of the bee-like structure; (**b**) the maximum height difference; (**c**) roughness; (**d**) the roughness of continuous phase).

Bitumen will age during use. This mainly occurs in the mixing, transportation, and paving of bituminous mixture. This short-term aging accounts for more than 80% of the total aging of the SBS modified bitumen life cycle [35]. Rolling thin-film oven test (RTFOT) is the most commonly used method for preparing short-term aged SBS modified bitumen. In this paper, the SBS modified bitumen after RTFOT was tested by AFM, and the nano-morphology parameters were analyzed. Figure 10 is the AFM images of bitumen with different SBS content after aging, and Figure 11 below is a comparison of the nano-parameters of SBS modified bitumen before and after RTFOT under different SBS content.

Figure 11 shows that the nano-morphology of SBS modified bitumen has changed to some extent after RTFOT. This is related to the aging of bitumen. During the aging process of bitumen, some aromatics is converted into resin, and part of the resin is converted into asphaltene. At the same time, some light components are volatilized, asphaltenes increase and aggregate. The changes make the average area of bee-like structure increase, while the height difference of bee-like structure itself fluctuates greatly, so the roughness of bitumen AFM image becomes larger, and the maximum height difference also increases correspondingly. In addition, the roughness of light components of SBS modified bitumen after aging is smaller than that before RTFOT, which may be due to the smoothing effect of SBS on light components.

**Figure 12:**
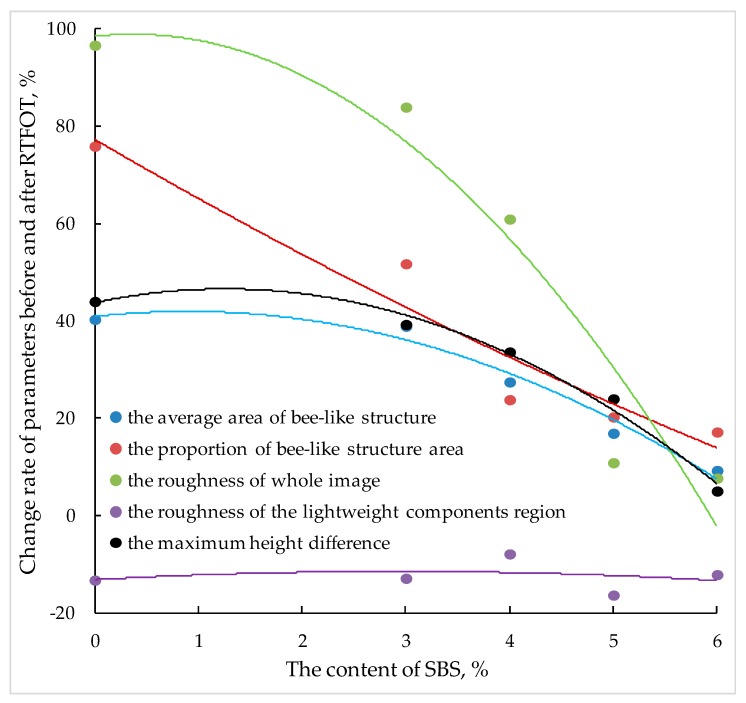
Increase of nano-parameters of SBS modified bitumen after RTFOT.

It can also be seen from Figure 11 that the changes of various nano-parameters of different SBS content before and after aging are different, which shows that the content of SBS affects the nano-morphology of SBS modified bitumen after aging to a certain extent. Figure 12 below shows the increase (decrease) of nano-parameters of SBS modified bitumen after short-term aging under different SBS content.

Figure 12 shows that the increase of nano-parameters of SBS modified bitumen after RTFOT is closely related to the content of SBS. With the increase of SBS content, the average area of the bee-like structure, the proportion of bee-like structure area, the roughness of the whole image, the maximum height difference, and other nano-parameters of modified bitumen show a decreasing trend, while the decrease of light component roughness is not obvious.

In conclusion, the larger the content of SBS, the smaller the influence of aging on the nano-morphology of SBS modified bitumen. According to the nanoscopic morphology characteristics of SBS modified bitumen at room temperature, as long as the asphaltene content increases, the percentage of bee-like structure area increases, therefore, asphaltene should be an important factor in the production of bee-like structures.

## 4. Relationship between Nano-Parameters and Pavement Performances

**Figure 13:**
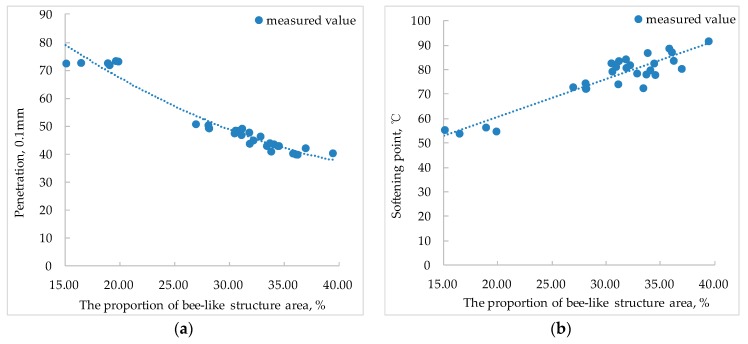
Relationship between the proportion of bee-like structure area and penetration, softening point of SBS modified bitumen ((**a**) penetration; (**b**) softening point).

**Figure 14:**
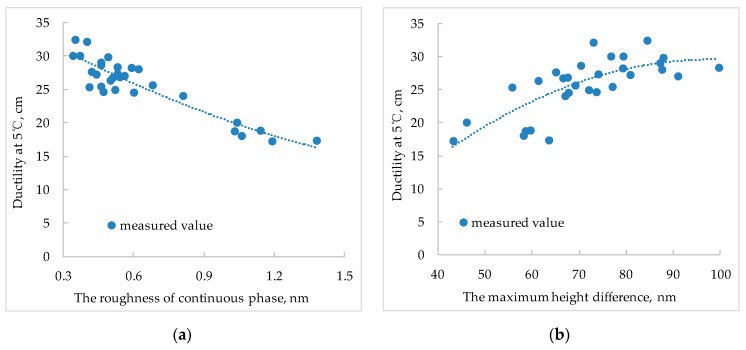
Relationship between the nano-parameters and ductility ((**a**) the roughness of continuous phase; (**b**) the maximum height difference).

**Figure 15:**
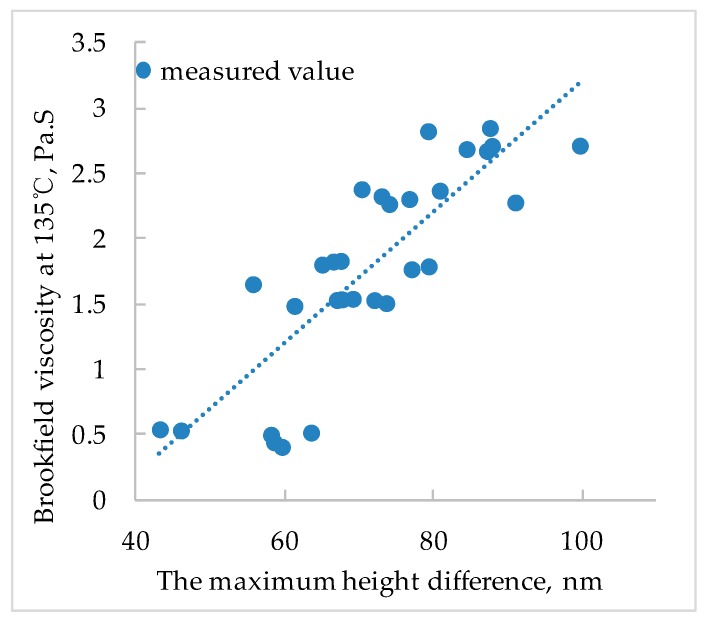
Relationship between Brookfield viscosity at 135 °C and the maximum height difference of SBS modified bitumen.

Establishing a functional relationship between nano-parameters and pavement performances of SBS modified bitumen is the fundamental purpose of studying nano-morphological characteristics. In this paper, the relationship between the technical indicators such as penetration, softening point, ductility, and viscosity of SBS modified bitumen and the nano-parameters was analyzed. Figure 13, Figure 14 and Figure 15 show the relationship between pavement performance and nano-parameters of SBS modified bitumen.

The penetration and softening point are the evaluation indicators of the bitumen viscosity. The smaller the penetration, the larger the viscosity of the bitumen, the larger the softening point, and the smaller the viscosity of the bitumen. Figure 13 shows that there is a good nonlinear relationship between the penetration of SBS modified bitumen and the proportion of bee-like structure area. As the proportion of bee-like structure area increases, the penetration of SBS modified bitumen decreases. The softening point of SBS modified bitumen has a linear relationship with the proportion of bee-like structure area. The larger the proportion of bee-like structure area, the higher the softening point. The mechanism of these phenomena is that the increase of the proportion of bee-like structure area means the increase of asphaltene, and the density of the asphaltene itself is larger, the hardness is higher, and the viscosity is larger, so that the viscosity of the SBS modified bitumen increases, resulting in the penetration being lowered and the softening point being increased.

After the SBS is mixed with bitumen, the asphaltene distribution is more uniform, which can improve the ductility of the SBS modified bitumen. In addition, the existence of SBS can reduce the roughness of light components and make the lighter components more uniform, which leads to the improvement of the bitumen ductility. In Figure 14a, as the roughness of the light component increases, the ductility of the SBS modified bitumen decreases gradually, which verifies the above analysis. In the AFM images of SBS modified bitumen, the maximum height difference is closely related to the nano-adhesion force of bitumen, and the larger the maximum height difference, the larger the nano-adhesion force. In addition, the nano-adhesion force is positively correlated with the low-temperature stiffness modulus of bitumen, i.e., the greater the maximum height difference, the higher the low-temperature stiffness modulus of bitumen, and the greater the ductility of bitumen.

Figure 15 shows that the greater the maximum height difference, the greater the Brookfield viscosity at 135 °C of the SBS modified bitumen. Since the maximum height difference is positively correlated with the content of SBS, the larger the SBS content, the larger the maximum height difference, the greater the Brookfield viscosity at 135 °C (SBS has a significant tackifying effect on bitumen). These reasons lead to a linear relationship between the Brookfield viscosity at 135 °C and the maximum height difference of the SBS modified bitumen.

In summary, there is a certain relationship between SBS modified bitumen nano-parameters and pavement performances. The bigger the proportion of bee-like structure area, the smaller the penetration, and the larger the softening point; the bigger the roughness of light component, the smaller the ductility; the bigger the maximum height difference, the greater the ductility; Brookfield viscosity at 135 °C of SBS modified bitumen presents a linear relationship with the maximum height difference.

## 5. Conclusions

In this paper, the nano-morphology of modified bitumen with different SBS content was tested by AFM and the related parameters were obtained. The relationship of nano-parameters with SBS content and the aging effect was analyzed, and the relationship between pavement performances of SBS modified bitumen and nano-morphology parameters was analyzed. The main conclusions are as follows:

(1) With the increase of the SBS content, the area of the bee-like structure of SBS modified bitumen and the proportion of bee-like structure area increases gradually and then tends to be stable, especially when the content of SBS is more than 4%, the increasing trend is no longer obvious. When the SBS content is 0%, the roughness of the whole image and the roughness of the continuous phase is bigger than that with other SBS contents. With the increase of SBS content, the roughness of whole image and the roughness of continuous phase show a decreasing trend, but when the SBS content exceeds 4%, the decreasing trend is no longer obvious.
(2) With the increasing of SBS content, the maximum height difference in the AFM image of SBS modified bitumen shows a linearly increasing trend.
(3) After the aging of SBS modified bitumen, the average area of the bee-like structure, the roughness, and the maximum height difference increases correspondingly, but the roughness of light components is smaller than that before aging
(4) The variation degree of nano-parameters before and after aging varies with different SBS content. The larger the content of SBS, the smaller the influence of aging on the nano-morphology of SBS modified bitumen.
(5) The bigger the proportion of bee-like structure area, the smaller the penetration, and the larger the softening point; the bigger the roughness of light component, the smaller the ductility; the bigger the maximum height difference, the greater the ductility; The Brookfield viscosity at 135 °C of SBS modified bitumen presents a linear relationship with the maximum height difference.

## Figures and Tables

**Table 1:** A-70 bitumen technical indicators.

Test Items	Unit	Measured Value	Test Items	Unit	Measured Value
5 °C Ductility	cm	19.1	Softening point	°C	55.1
135 °C Brookfield viscosity	Pa·s	0.493	15 °C Density	g/cm^3^	0.999
15 °C Ductility	cm	>98	25 °C Penetration	0.1 mm	72.8
RTFOT	Penetration ratio			%	72.9
	Loss of quality			%	0.5
	5 °C Residue Ductility			cm	11.3

RTFOT: Rolling Thin Film Oven Test.

**Table 2:** Test conditions of AFM.

Test Conditions	Value
Test Temperature	25 °C
Scan Size	10 × 10 μm
Scan Rate	2.5 Hz
Number of Samples	265
Amplitude Setpoint	731.34 mV
Drive Amplitude	300.00 mV
